# Phylogenetic Meta-Analysis of the Functional Traits of Clonal Plants Foraging in Changing Environments

**DOI:** 10.1371/journal.pone.0107114

**Published:** 2014-09-12

**Authors:** Xiu-Fang Xie, Yao-Bin Song, Ya-Lin Zhang, Xu Pan, Ming Dong

**Affiliations:** 1 Key Laboratory of Hangzhou City for Ecosystem Protection and Restoration, College of Life and Environmental Sciences, Hangzhou Normal University, Hangzhou, China; 2 State Key Laboratory of Vegetation and Environmental Change, Institute of Botany, Chinese Academy of Sciences, Beijing, China; University of Leipzig, Germany

## Abstract

Foraging behavior, one of the adaptive strategies of clonal plants, has stimulated a tremendous amount of research. However, it is a matter of debate whether there is any general pattern in the foraging traits (functional traits related to foraging behavior) of clonal plants in response to diverse environments. We collected data from 97 published papers concerning the relationships between foraging traits (e.g., spacer length, specific spacer length, branch intensity and branch angle) of clonal plants and essential resources (e.g., light, nutrients and water) for plant growth and reproduction. We incorporated the phylogenetic information of 85 plant species to examine the universality of foraging hypotheses using phylogenetic meta-analysis. The trends toward forming longer spacers and fewer branches in shaded environments were detected in clonal plants, but no evidence for a relation between foraging traits and nutrient availability was detected, except that there was a positive correlation between branch intensity and nutrient availability in stoloniferous plants. The response of the foraging traits of clonal plants to water availability was also not obvious. Additionally, our results indicated that the foraging traits of stoloniferous plants were more sensitive to resource availability than those of rhizomatous plants. In consideration of plant phylogeny, these results implied that the foraging traits of clonal plants (notably stoloniferous plants) only responded to light intensity in a general pattern but did not respond to nutrient or water availability. In conclusion, our findings on the effects of the environment on the foraging traits of clonal plants avoided the confounding effects of phylogeny because we incorporated phylogeny into the meta-analysis.

## Introduction

Essential resources such as light, water and soil nutrients often have heterogeneous distributions in natural habitats [1]-[4]. Phenotypic plasticity is an adaptive strategy through which plants can cope with environmental variation in space and time [5], [6]. Clonal plants occur in many different taxonomic groups [7] and are dominant in many natural and man-made ecosystems [7], [8]. The success of clonal plants may occur because their distinctive life-history strategies [9], [10] allow them to cope with the heterogeneity of essential resources. One of these strategies is plastic foraging, i.e., the processes whereby an organism searches or ramifies within its habitat to enhance its acquisition of essential resources [6], [11], [12]. Foraging strategies help clonal plants escape from unfavorable patches and/or exploit favorable ones by altering the clonal morphology of spacers and branching in patchy environments [6], [13], [14].

The adaptive evolution of clonal life history traits may be limited by physiological or physical constraints [10], [15]. Such constraints may limit the plastic foraging [16] responses of species to environmental change [17]. For example, because of their different functions, stolons might be more plastic than rhizomes in response to a light resource, whereas rhizomes might be more plastic than stolons in response to a nutrient resource [18]. The results of a garden experiment showed that plants with contrasting branching patterns (monopodial versus sympodial) exhibited different foraging traits [17]. Therefore, we may expect the process of plastic foraging to differ among species with different clonal-organ types (i.e., stoloniferous and rhizomatous) and branching-form types (i.e., monopodial and sympodial). The process of plastic foraging of clonal plants may also depend on the response of plants to resources (i.e., water, nutrients or carbohydrates). However, this hypothesis has not been tested because multiple species are involved.

To achieve efficient plastic foraging, clonal plants usually adopt two tactics: branching and spacing. Ramets can regulate the plasticity of branching or spacing in patches with high- or low-level resources [19]. Branching traits include the plasticity of branching intensity (i.e., the number of branches per ramet or node of the rhizome or stolon [13]) and branching angle (i.e., the angle between adjacent branches in the horizontal plane [14]). Spacing traits include the plasticity of spacer length (i.e., the distance between adjacent ramets, which may contain only one internode [13]) and spacer thickness (i.e., specific spacer length, which is the ratio of the spacer length to the dry mass of the spacer). The trade-off between branching and spacing as plastic foraging strategies may be determined by environmental factors [19]. Thus far, whether branching traits are more plastic than spacing traits has not been tested.

A meta-analytic approach is optimal to analyze the responses of clonal plants to various environments (i.e., [20], [21]). However, Adams [22] stated that traditional meta-analytic approaches lacked independence because of species with shared evolutionary histories in biology and ecology. Adams [22] proposed that the phylogenetic meta-analysis (PMA) model that incorporates phylogenetic information into the traditional meta-analysis be used. Furthermore, a recent meta-analysis demonstrated that incorporating phylogenetic information significantly changed the pooled effect sizes of traditional meta-analysis [23].

We adopted the phylogenetic meta-analytic method proposed by Lajeunesse [24] to answer the following three questions for clonal plants: 1) Are branching traits more plastic than spacing traits in response to resource availability? 2) Does the foraging behavior of clonal plants differ with different clonal architectures? 3) Do the foraging-related traits depend on the type of resource?

## Materials and Methods

### Literature survey and data selection criteria

**Figure 1:**
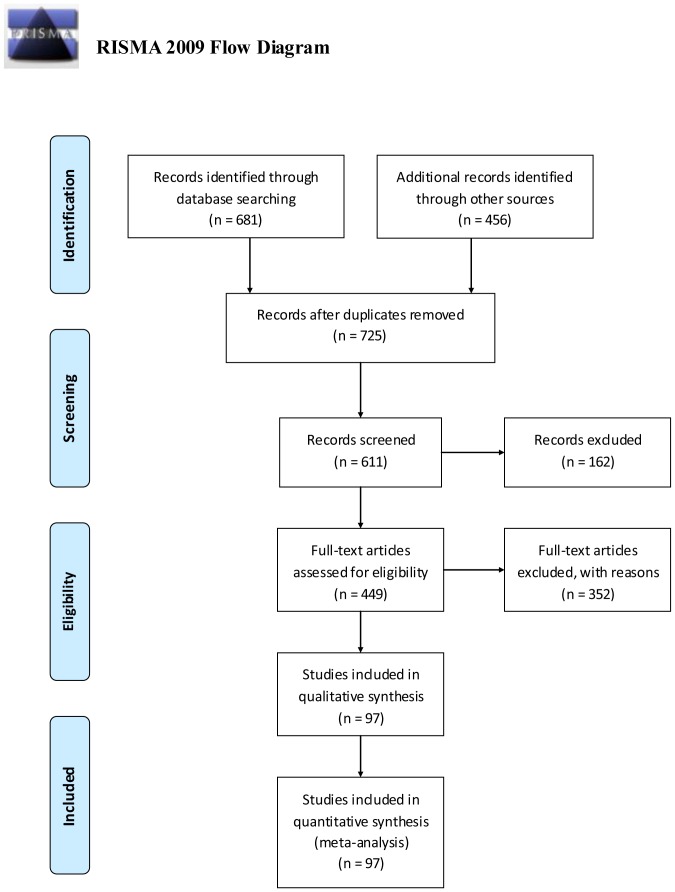
The flow diagram.

To be comprehensive and to avoid bias in the literature survey, we conducted an exhaustive, strategic search of all literature concerning our scientific questions, relying primarily on the internet search engine Google Scholar, which covers most peer-reviewed papers, theses and dissertations (or degree papers), books, and other published or unpublished academic literature from broad areas of research [25]. To identify studies specific to our questions, the survey was supplemented by additional searches of a number of main databases (e.g., ISI Web of Knowledge). We restricted the search keywords to papers whose topic referred to “forag*”, “spacer length”, “rhizome length”, “stolon length” or “internode length” in combination with “clonal plant”. We obtained 715 published papers from which we selected 449 studies as suitable for the meta-analysis (Figure 1; [26]).

For each publication, we recorded the title, author(s), year, location, and other information (see Appendix S1) and examined its potential for meeting the selection criteria for inclusion in the review. Furthermore, only the publications that reported values of plant clonal traits for both the treatment and control groups in (greenhouse, garden or field) experiments were considered, whereas reviews, publications on models and other studies were excluded. In our meta-analysis, we only included studies that reported traits related to foraging strategy (e.g., spacing traits, branching traits) in response to resource availability (Table 1). We classified the resources into three categories (Table 2)—light intensity, nutrient level and water availability—which are usually heterogeneously distributed in nature. Thus, other resources, such as CO_2_ [26], were not considered for this review. Furthermore, we excluded the studies in which the means for the treatment and control groups were not reported with the sample size and/or the standard deviation or in which we were unable to infer (or calculate) the sample size or standard deviation from other information provided in the study [27]. Our final data set contained 97 papers published from 1965 to 2013 in 37 journals and provided the data for the meta-analyses (Appendix S2).

**Table 1:** Foraging traits and their subcategories and categories (foraging tactics).

Foraging tactics	Foraging trait subcategory	Foraging trait
Spacing	Spacer length (SL)	Rhizome length, spacer length, stolon length.
	Specific spacer length (SSL)	Specific rhizome length, specific spacer length, specific stolon length.
Branching	Branching intensity (BI)	Branching index, branching intensity, number of branches, number of rhizomes, number of stolons.
	Branching angle (BA)	Branching angle.

**Table 2:** Resource treatments used in the studies reported in the literature.

Resource category	Treatments
Light	Darkening (-), light, light increased, low-light (-), partial shading (-), shade (-), shading (-).
Nutrients	Fertilization, litter, low-N (-), N, nutrient, P, sediment type, soil, soil nutrient, soil resources.
Water	Drought (-), low-water (-), soil moisture, soil water, water, water amount, water reduced (-), wet treatment.

(-): Opposite direction of the treatment effect.

### Data assembly

From each study, we extracted the mean, a measure of statistical variation (usually standard error or standard deviation) and the sample size for the treatment and control groups for each response variable. We regarded multiple results within a single paper as different results from independent studies when they involved different species and/or treatments [27]-[31]. We only extracted data once when the same experimental results were published in different papers [32]. To collect original data, the graphs in articles were digitized with GetData (Graph Digitizer v2.22 Datatrend Software, Raleigh, North Carolina, USA) [33], and digitized values were accurate to ±1% of the actual value [27], [29].

A total of 1,370 comparisons encompassing 85 clonal plant species from 64 genera belonging to 28 families met our criteria. For each comparison, we calculated Hedges' *d* as a measure of the effect magnitude because it is the preferred measure of effect size for meta-analysis and because it has a lower Type I error rate than other measures, such as the log-response ratio [34], [35]. The absolute value shows the magnitude of the treatment impact. Positive and negative *d* values signify an increase and a decrease in the effect of the treatment, respectively. A value of zero indicates no difference between the treatment and control groups.

For studies that described experiments with several treatment levels or times, we pooled the effect sizes and variances for each response variable of each species in a study, and we conducted a separate meta-analysis on all traits and treatments of the respective trait category to avoid pseudo-replication (see also [34], [35]). The estimated pooled mean effect size and the mean variance were used in the final data sets containing 107 pairwise comparisons with light treatments, 179 pairwise comparisons with nutrient treatments and 26 pairwise comparisons with water treatments (Appendix S1). To pool the effect sizes and all analyses, we chose the random-model approach because we assumed that the differences among comparisons and among studies were not only due to sampling error but also to true random variation, as is the rule for ecological data [36]. All the effect size calculations and pooling were performed with Metawin software, version 2.1 [37].

To apply PMA, we first created a phylogeny including all the plant species using the Phylomatic software online (http://phylodiversity.net/phylomatic/; with option Phylomatic tree R20120829 for plants). The branch length for the phylogeny was estimated using the ‘bladj’ function in the ‘Phylocom’ software [38], [39] (the constructed phylogeny with branch length is provided in Appendix S3). Using the same procedure, we generated a subset phylogeny for each trait category including the corresponding subset species pool. The branch length was again estimated using the age file in Phylocom software. Because of the restriction of input files executed on phyloMeta v1.3 software [40], we again pooled those multiple effect sizes for the same species. The result was one accumulated weighted effect size and variance for each species within a given trait category. However, this approach inevitably resulted in smaller sample sizes for each trait category (N_effect-size_ =  N_species_) [41], [42]. The pooling was also conducted with a random-effects model on Metawin, version 2.1 [37].

### Data analysis

An inherent problem with meta-analysis is the potential for publication bias, which has been termed the “file-drawer problem” [43]. Therefore, before all analyses, we explored the possibility of publication bias graphically (using a funnel plot and normal quantile plot) [44], [45], statistically (using the Spearman rank correlation test) [46] and by calculating a fail-safe number [47], [48], which is the number of studies that would have to be added to change the results of the meta-analysis from significant to nonsignificant [49].

For each trait category (Table 1), we calculated the overall effect sizes (*d+*) of every resource category (Table 2) separately across the samples of case studies with information on the relevant response variables. The overall effect sizes were cumulative effect sizes per species [24], [50]. For the interspecific differences in spacer length, we conducted a supplementary analysis on its two determinants—internode length and node number—because a spacer could have more internodes, in addition to a single-internode spacer. To detect the differences between stoloniferous and rhizomatous plants and between monopodial and sympodial plants, we considered clonal organ type (i.e., stoloniferous versus rhizomatous) and branching form type (i.e., monopodial versus sympodial) as moderator variables [41]. The analyses were performed with the software phyloMeta v1.3 [41].

## Results

### Generalities of sampled studies

The overall data exploration found no evidence of publication bias. The funnel plot of effect size versus sample size showed no skewness (Appendix S3). A plot of the standardized effect sizes against the normal quantiles revealed a straight line (Appendix S3). These two graphical approaches suggest that there was no bias in the results from this meta-analysis. This result was further emphasized by a nonsignificant result of the Spearman rank-order correlation test (R  =  -0.091, *p*> 0.05). Finally, the weighted fail-safe number 10,139 was much greater than expected (5n + 10  =  1570) without publication bias, which supports the robustness of our results. Thus, we are confident that our results provide reliable estimates of the true effects.

### PMA on foraging responses of clonal plants to resource heterogeneity

**Figure 2:**
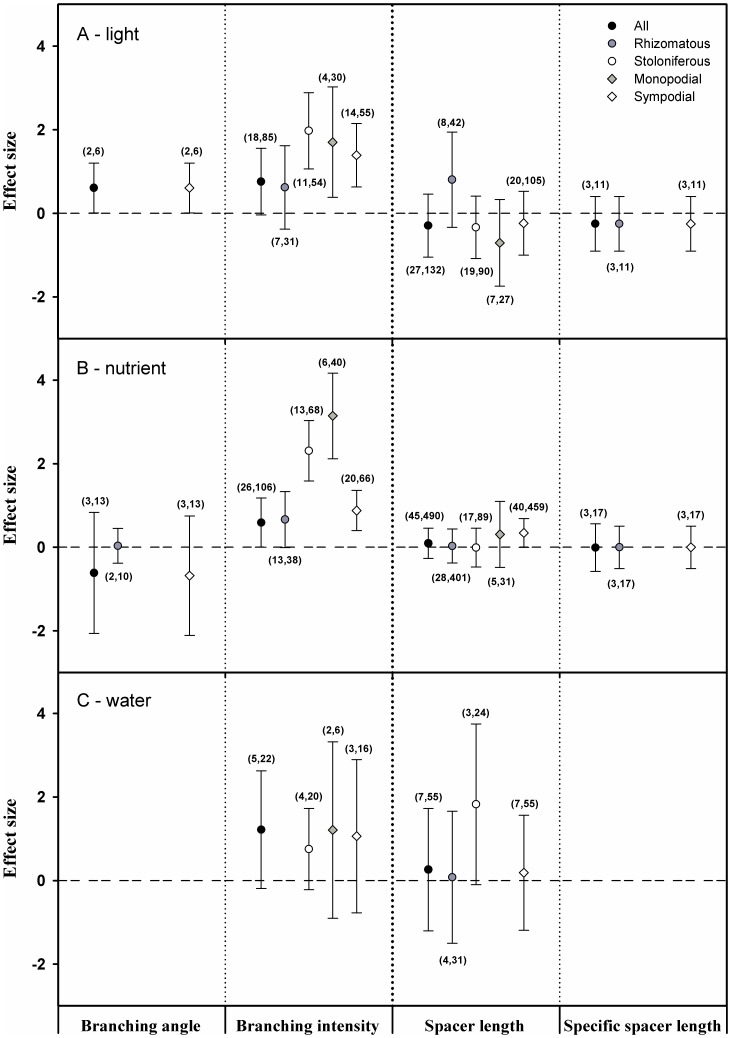
The results from PMA using phyloMeta v1.3. Black circle: the mean effect size of the three types of resources (A: light; B: nutrients; C: water) on all plants. Gray circle: the mean effect size of the three types of resources on rhizomatous plants. White circle: the mean effect size of the three types of resources on stoloniferous plants. Gray diamond: the mean effect size of the three types of resources on monopodial plants. White diamond: the mean effect size of the three types of resources on sympodial plants. Dotted line: the reference line of an effect size equal to zero. The numbers in parentheses: the first is the number of species contained, and the second is the number of cases combined. *BA: branching angle; BI: branching intensity; SL: spacer length; SSL: specific spacer length.

In the PMA results, the grand mean effect sizes of light on spacing traits (spacer length and specific spacer length) and branching traits (branching intensity) were not significant except for that on branching angle (*d+*  =  0.61, N  =  2, 95% CI  =  0.01 to 1.20; Figure 2A). The grand mean effect sizes of nutrients on spacing traits and branching traits were not significant except for that on branching intensity (*d+*  =  0.59, N  =  26, 95% CI  =  0.01 to 1.18; Figure 2B). The grand mean effect sizes of water on spacing traits and branching traits were not significant.

For the clonal organ type, the spacing traits were not significantly different from zero when responding to light in either stoloniferous plants or rhizomatous plants (Figure 2A). The branching intensity response to light was significant for the stoloniferous plants (*d+* =  1.97, N  =  11, 95% CI  =  1.06 to 2.89), but it was not significant for the rhizomatous plants (Figure 2A). The branching intensity of the stoloniferous plants was significantly greater than zero when responding to nutrient level (*d+* =  2.31, *N* =  13, 95% CI  =  1.59 to 3.03), whereas the branching intensity of rhizomatous plants was not significant (Figure 2B). The effect sizes of nutrients on the branching intensity of stoloniferous and rhizomatous were significantly different (*Q_b_* =  11.93, *P* < 0.05). The effect sizes of nutrients on the branching intensity of monopodial and sympodial plants were also significantly different (*Q_b_* =  69.66, *P* < 0.05). However, nutrients had no effects on the spacing traits or the branching angle for either the stoloniferous or the rhizomatous plants (Figure 2B). Moreover, no significant effects of water on spacing traits or branching traits were detected (Figure 2C).

For the branching forms, spacing traits were not significantly different from zero when responding to light either in monopodial or sympodial plants (Figure 2A). The branching traits of both the monopodial and sympodial plants were significantly different from zero when responding to light, except for the branching angle of the monopodial plants for which data were lacking (Figure 2A). Similarly, spacing traits were not significantly different from zero when responding to nutrient level in either the monopodial or the sympodial plants (Figure 2B). The branching intensity of the monopodial and sympodial plants was significantly different from zero when responding to nutrient level (Figure 2B). Neither spacing traits nor branching traits were significantly different from zero when the monopodial and sympodial plants responded to water.

### PMA on the responses of internodes to resource heterogeneity

**Figure 3:**
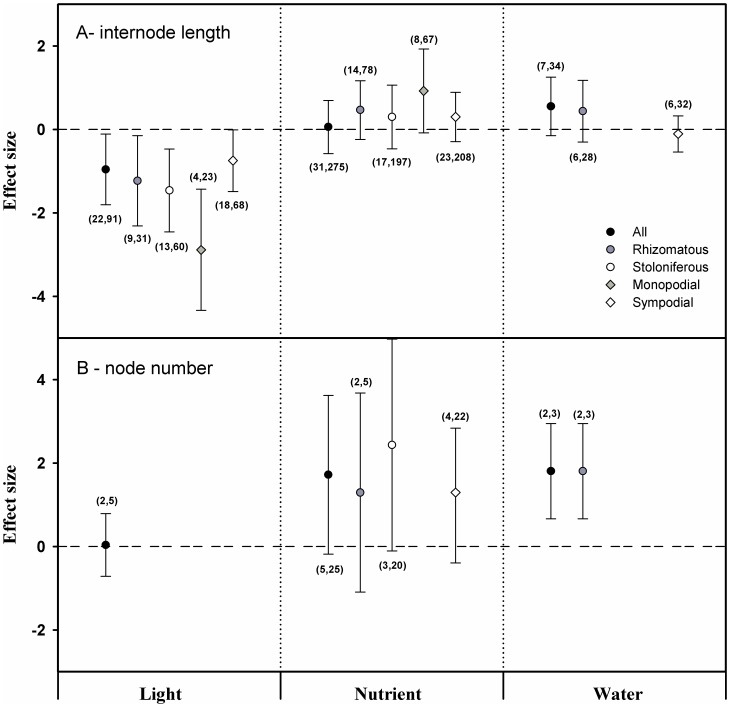
The results from the supplementary analyses (A: internode length; B: node number) with the random-model in PMA using phyloMeta v1.3. Black circle: the mean effect size of the three types of resources on all plants. Gray circle: the mean effect size of the three types of resources on rhizomatous plants. White circle: the mean effect size of the three types of resources on stoloniferous plants. Gray diamond: the mean effect size of the three types of resources on monopodial plants. White diamond: the mean effect size of the three types of resources on sympodial plants. Dotted line: the reference line of an effect size equal to zero. The numbers in parentheses: the first is the number of species contained, and the second is the number of cases combined. *IL: internode length; NN: node number.

According to the supplementary analyses, the internode length was significantly less than zero when responding to light but was not significantly different from zero when responding to nutrients or water, regardless of the clonal organ type or branching form (Figure 3A). Neither light nor nutrients exerted any significant effects on the node number, but water did have positive effects on the node number (Figure 3B).

Additionally, the results of all effects on the specific space length, branching angle and node number should be interpreted with caution because of the limited number of species and should therefore only be treated as a reference point (Figure 2).

## Discussion

### Responses of foraging traits to resource heterogeneity

Clonal plants adapt to changing environments by developing different adaptive strategies, mainly in the plasticity of plant traits. Our analyses provided powerful evidence that clonal plants indeed adopted foraging strategies in response to diverse environments [6], [51]. Under shaded conditions, clonal plants decreased their branching intensity and tended to increase their spacer length to seek light resources, especially stoloniferous plants. This result was consistent with previous findings from empirical, experimental [52]-[55] and model [12], [56] studies. However, we found that internode length was more flexible in response to light intensity, whereas spacer length had no significant response to environmental heterogeneity. One possible explanation for these findings is that clonal plants may produce spacers with shorter internodes and more nodes under high light conditions, which is supported by the results of our analyses. Thus, the spacer length did not show any significant elongation under shaded conditions. Based on empirical and theoretical studies, another spacing variable (specific spacer length) should increase in response to shading because of limited biomass production in shaded environments [10], [57]. However, few studies have focused on the plasticity of specific spacer length, which should be examined in the future.

Our analyses showed that nutrient availability had little effect on spacing traits or branching angle, but it did have a positive effect on branching intensity, even though this effect varied with different clonal architectures. Our findings support previous research that found that with increasing nutrient availability, branching intensity increased. The branch angle did not exhibit any significant plasticity, and the response of spacer length was species specific [58]. One interpretation may be that the elongation and maintenance of spacer length comes with a cost, but the plant cannot pay this cost when the nutrient availability is too low [59]-[61], implying that there might be a trade-off between spacing and branching strategies. A significant impact of water on spacer length was not detected in our study. Unfortunately, the relationship between water availability and the other two traits was not clear and the results were not definitive because of limited data. At present, experimental research on the morphological plasticity of clonal plants in response to water availability is relatively lacking, and there is a particular need for such studies (see the example in [62]).

### Differences between stoloniferous and rhizomatous plants in foraging tactics

Our results suggested that the tactics used for foraging for light resources were distinct for stoloniferous and rhizomatous species [63]. For stoloniferous plants, light had an appreciably negative effect on internode length and a positive effect on branching intensity. These observations were consistent with foraging theory. However, for rhizomatous plants, there were no significant relationships for light and foraging traits except for the internode length. Therefore, as generally accepted [18], [64], [65], stoloniferous plants were more sensitive to light than rhizomatous plants. An unexpected result was that we did not find a greater impact of nutrients on the rhizomatous plants compared with the stoloniferous plants, as recognized previously by Dong & de Kroon [18]. However, nutrients had no significant impacts on foraging traits except for the branching intensity of stoloniferous plants. For the rhizomatous plants, increasing nutrient availability had no obvious impact on any foraging trait. This variation in response might be due to the different functions of the different organs; rhizomes serve as organs primarily for the storage of resources and meristems, whereas stolons serve as organs primarily for foraging [18], [66]. The data on the effects of water on foraging traits were limited and did not allow us to determine how water influenced the foraging behaviors of clonal plants.

Additionally, our results indicated that the foraging tactics used by monopodial and sympodial plants were similar, with both decreasing their branching intensity under resource-poor conditions and lengthening their internodes under shaded conditions. Thus, the foraging behaviors of clonal plants do not vary with branching form.

Meta-analysis has become a common method in ecological studies, and phylogenetic information is incorporated into meta-analyses with increasing regularity. However, phylogenetic meta-analysis is still in its infancy for use in clonal plant research. In our study, we conducted a parallel analysis for comparison with the traditional meta-analysis method. The results indicated that the incorporation of phylogenetic information into the analyses across our data sets slightly altered the significance of some effect sizes (Appendix S4). Moreover, the phenotype (trait) of a species is derived from the combined effects of local environments and genetic factors, and the genetic factor reflects the evolutionary history (phylogenetic information) [17]. PMA provided us with more convincing results than the traditional meta-analysis because we avoided the confounding influence of genetics and eliminated the possibility that closely related species might have similar responses to a changing environment.

In conclusion, our study is the first to use PMA to analyze the responses of the foraging traits of clonal plants to heterogeneous environments. We summarize the general patterns of our PMA analysis as follows: 1) clonal plants exhibit a higher plasticity of foraging traits in response to light intensity than to nutrient level or water availability; 2) spacer length, and sometimes internode length, is more flexible in response to light heterogeneity, and branch intensity is more sensitive to nutrient heterogeneity; and 3) stoloniferous plants show much stronger morphological plasticity in terms of foraging traits than rhizomatous plants. In this paper, we only tested the foraging hypotheses and aimed to clarify the general patterns of foraging behavior-related traits in clonal plants, but the mechanisms underlying these patterns must be explored in future research.

## Supporting Information

Appendix S1 General information and data table with the details from all the single studies.
(XLS)

Appendix S2 List of the studies used for meta-analysis in this article.
(DOC)

Appendix S3 Phylogenetic tree, funnel plot and normal quantile plot.
(DOC)

Appendix S4 Results with the random-model in traditional meta-analysis versus PMA by phyloMeta v1.3.
(DOC)
