# Supplementary material for: Phylogenetic Meta-Analysis of the Functional Traits of Clonal Plants Foraging in Changing Environments
Source: PLoS One. 2014 Sep 12;9(9):e107114. doi: 10.1371/journal.pone.0107114 (PMC4162570; doi:10.1371/journal.pone.0107114)
Supplement: Appendix S2 — List of the studies used for meta-analysis in this article. (DOC) [file pone.0107114.s002.doc]

**Appendix B:** List of studies in this meta-analysis.

Alpert P (1991) Nitrogen sharing among ramets increases clonal growth in *Fragaria chiloensis*. Ecology 72: 69-80.

Ba L, Wang DL, Hodgkinson KC, Xiao NZ (2006) Competitive relationships between two contrasting but coexisting grasses. Plant Ecology 183: 19-26.

Ba L, Xiao NZ, Xu W, Wang DL (2008) Effects of nutrient availability and competition between two grass species *Leymus chinensis* (Trin.) Tzvel. and *Phragmites communis* Trin. on the plant performance. Polish Journal of Ecology 56: 453-462.

Balestri E, Vallerini F, Lardicci C (2010) Effect of seed density and sediment nutrient heterogeneity on recruitment and early patch growth in the seagrass *Cymodocea nodosa*. Marine Ecology Progress Series 417: 63-72.

Chen JS (2004) Study on the adaptive strategies of clonal plants in heterogeneous environments. Wuhan: Wuhan University. 118 p.

Claridge K, Franklin SB (2002) Compensation and plasticity in an invasive plant species. Biological Invasions 4: 339-347.

Clevering OA (1999) The effects of litter on growth and plasticity of *Phragmites australis* clones originating from infertile, fertile or eutrophicated habitats. Aquatic Botany 64: 35-50.

Collins RP, Fothergill M, Macduff JH, Puzio S (2003) Morphological compatibility of white clover and perennial ryegrass cultivars grown under two nitrate levels in flowing solution culture. Annals of Botany 92: 247-258.

Cronin G, Lodge DM (2003) Effects of light and nutrient availability on the growth, allocation, carbon/nitrogen balance, phenolic chemistry, and resistance to herbivory of two freshwater macrophytes. Oecologia 137: 32-41.

D'Hertefeldt T, Falkengren-Grerup U, Jonsdottir IS (2011) Responses to mineral nutrient availability and heterogeneity in physiologically integrated sedges from contrasting habitats. Plant Biology 13: 483-492.

de Kroon H, Whigham DF, Watson MA (1991) Developmental ecology of mayapple: Effects of rhizome severing, fertilization and timing of shoot senescence. Functional Ecology 5: 360-368.

Dong M (1993) Morphological plasticity of the clonal herb *Lamiastrum galeobdolon (*L.) Ehrend. & Polatschek in response to partial shading. New Phytologist 124: 291-300.

Dong M (1995) Morphological responses to local light conditions in clonal herbs from contrasting habitats, and their modification due to physiological integration. Oecologia 101: 282-288.

Dong M, Alaten B (1999) Clonal plasticity in response to rhizome severing and heterogeneous resource supply in the rhizomatous grass *Psammochloa villosa* in an Inner Mongolian dune, China. Plant Ecology 141: 53-58.

Dong M, During HJ, Werger MJA (1996) Morphological responses to nutrient availability in four clonal herbs. Vegetatio 123: 183-192.

Dong M, During HJ, Werger MJA (1997) Clonal plasticity in response to nutrient availability in the pseudoannual herb, *Trientalis europaea* L. Plant Ecology 131: 233-239.

Dong M, During HJ, Werger MJA (2002) Root and shoot plasticity of the stoloniferous herb *Ajuga reptans* L. planted in a heterogeneous environment. Flora 197: 37-46.

Dong M, Pierdominici MG (1995) Morphology and growth of stolons and rhizomes in three clonal grasses, as affected by different light supply. Plant Ecology 116: 25-32.

Dong M, Zhang SM, Chen YF (2000) Clonal plasticity in response to nutrient availability in the stoloniferous herb, *Duchesnea indica*. Acta Botanica Sinica 42: 518-522.

Eriksson O (1986) Mobility and space capture in the stoloniferous plant *Potentilla anserina*. Oikos 46: 82-87.

Fan YN (2009) Phenotypic plasticity and spatial expansion strategy of *Potentilla anserine* clonal population in heterogeneity habitat. Jilin: Northeast Normal University. 40 p.

Gianoli E (2001) Lack of differential plasticity to shading of internodes and petioles with growth habit in *Convolvulus arvensis* (Convolvulaceae). International Journal of Plant Sciences 162: 1247-1252.

Ginzo HD, Lovell PH (1973) Aspects of the comparative physiology of *Ranunculus bulbosus* L. and *Ranunculus repens* L. I. Response to nitrogen. Annals of Botany 37: 753-764.

González AV, Gianoli E (2004) Morphological plasticity in response to shading in three *Convolvulus* species of different ecological breadth. Acta Oecologica 26: 185-190.

He Z, He W, Yu F, Shi P, Zhang X, et al. (2007) Do clonal growth form and habitat origin affect resource-induced plasticity in Tibetan alpine herbs? Flora 202: 408-416.

Holdredge C, Bertness MD, von Wettberg E, Silliman BR (2010) Nutrient enrichment enhances hidden differences in phenotype to drive a cryptic plant invasion. Oikos 119: 1776-1784.

Huber-Sannwald E, Pyke DA, Caldwell MM, Durham S (1998) Effects of nutrient patches and root systems on the clonal plasticity of a rhizomatous grass. Ecology 79: 2267-2280.

Huber H (1996) Plasticity of internodes and petioles in postrate and erect *Potentilla* species. Functional Ecology 10: 401-409.

Huber H, Fijan A, During HJ (1998) A comparative study of spacer plasticity in erect and stoloniferous herbs. Oikos 81: 576-586.

Huber H, Wiggerman L (1997) Shade avoidance in the clonal herb *Trifolium fragiferum*: A field study with experimentally manipulated vegetation height. Plant Ecology 130: 53-62.

Huffman DW, Zasada JC, Tappeiner Ii JC (1994) Growth and morphology of rhizome cuttings and seedlings of salal (*Gaultheria shallon*): Effects of four light intensities. Canadian Journal of Botany 72: 1702-1708.

Hutchings MJ, Turkington R, Carey P, Klein E (1997) Morphological plasticity in *Trifolium repens* L.: The effects of clone genotype, soil nutrient level, and the genotype of conspecific neighbours. Canadian Journal of Botany 75: 1382-1393.

Ikegami M, Whigham D, Werger M (2007) Responses of rhizome length and ramet production to resource availability in the clonal sedge *Scirpus olneyi* A. Gray. Plant Ecology 189: 247-259.

Ikegami M, Whigham D, Werger M (2009) Ramet phenology and clonal architectures of the clonal sedge *Schoenoplectus americanus* (Pers.) Volk. ex Schinz & R. Keller. Plant Ecology 200: 287-301.

Jensen S, Bell S (2001) Seagrass growth and patch dynamics: Cross-scale morphological plasticity. Plant Ecology 155: 201-217.

Jongejans E, de Vere N, de Kroon H (2008) Demographic vulnerability of the clonal and endangered meadow thistle. Plant Ecology 198: 225-240.

Kang XY (2007) Study on the growth character in the clonal plant: *Potentilla anserine* L. Xining: Qinghai University. 49 p.

Kemball WD, Palmer MJ, Marshall C (1992) The effect of local shading and darkening on branch growth, development and survival in *Trifolium repens* and *Galium aparine*. Oikos 63: 366-375.

Kleijn D, Van Groenendael JM (1999) The exploitation of heterogeneity by a clonal plant in habitats with contrasting productivity levels. Journal of Ecology 87: 873-884.

Landhäusser SM, Stadt KJ, Lieffers VJ, McNabb DH (1996) Rhizome growth of *Calamagrostis canadensis* in response to soil nutrients and bulk density. Canadian Journal of Plant Science 76: 545-550.

Li B, Shibuya T, Yogo Y, Hara T, Matsuo K (2001) Effects of light quantity and quality on growth and reproduction of a clonal sedge, *Cyperus esculentus*. Plant Species Biology 16: 69-81.

Li DZ, Shigeo T, Zhu TC (2006) Effects of clonal connection and segmentation on the primary stolons of *Zoysia japonica* in environments with heterogeneous soil nitrogen resources. Acta Prataculturae Sinica 15: 115-123.

Li QY (2006) Effects of different soil nutrients on clonal growth and sexual reproduction in *Iris Japonica* Thunb. Chongqing: Southwest University. 47 p.

Liao YM, Lei NF, Chen JS (2006) Clonal integration of a stoloniferous plant (*Fragaria vesca*) in response to heterogeneous light. Guihaia 26: 503-506.

Liu JM (2010) Study on the ecological characteristics of *Drepanostachyum luodianense* in Guizhou karst area. Beijing: Beijing Forestry University. 155 p.

Liu Q, Li YX, Zhong ZC (2004) Effects of moisture availability on clonal growth in bamboo *Pleioblastus maculata*. Plant Ecology 173: 107-113.

Liu Q, Zhong ZC (1996) The effects of water resources supply on clonal growth in *Pleioblastus maculata* population. Acta Phytoecologica Sinica 3: 245-254.

Liu Y, Schieving F, Stuefer JF, Anten NP (2007) The effects of mechanical stress and spectral shading on the growth and allocation of ten genotypes of a stoloniferous plant. Annals of Botany 99: 121-130.

Louapre P, Bittebiere AK, Clement B, Pierre JS, Mony C (2012) How past and present influence the foraging of clonal plants? PLoS One 7: e38288.

Luo XG, Dong M (2001) Architectural plasticity in response to light intensity in the stoloniferous herb, *Duchesnea indica* Focke. Acta Phytoecologica Sinica 25: 494-497.

Luo XG, Dong M (2001) Plasticity of clonal architecture in response to soil nutrients in the stoloniferous herb *Duchesnea indica* Focke. Acta Ecologica Sinica 21: 1957-1963.

Méthy M, Alpert P, Roy J (1990) Effects of light quality and quantity on growth of the clonal plant *Eichhornia crassipes*. Oecologia 84: 265-271.

Macdonald SE, Lieffers VJ (1993) Rhizome plasticity and clonal foraging of *Calamagrostis canadensis* in response to habitat heterogeneity. Journal of Ecology 81: 769-776.

Macek P, RejmÁNkovÁ E (2007) Response of emergent macrophytes to experimental nutrient and salinity additions. Functional Ecology 21: 478-488.

Marshall C, Anderson-Taylor G (1992) Mineral nutritional inter-relations amongst stolons and tiller ramets in *Agrostis stolonifera* L. New Phytologist 122: 339-347.

McIntyre GI (1965) Some effects of the nitrogen supply on the growth and development of *Acropyron repens* L. Beauv. Weed Research 5: 1-12.

Miao SL (2004) Rhizome growth and nutrient resorption: Mechanisms underlying the replacement of two clonal species in Florida Everglades. Aquatic Botany 78: 55-66.

Navas ML, Garnier E (2002) Plasticity of whole plant and leaf traits in *Rubia peregrina* in response to light, nutrient and water availability. Acta Oecologica 23: 375-383.

Niva M, Svensson BM, Karlsson PS (2006) Effects of light and water availability on shoot dynamics of the stoloniferous plant *Linnaea borealis*. Ecoscience 13: 318-323.

Olga A C (1998) An investigation into the effects of nitrogen on growth and morphology of stable and die-back populations of *Phragmites australis*. Aquatic Botany 60: 11-25.

Piqueras J, Klimes L, Redbo-Torstensson P (1999) Modelling the morphological response to nutrient availability in the clonal plant *Trientalis europaea* L. Plant Ecology 141: 117-127.

Schmid B, Bazzaz FA (1992) Growth-responses of rhizomatous plants to fertilizer application and interference. Oikos 65: 13-24.

Shan BQ, Du GZ, Liu ZH (2000) Clonal growth of *Ligularia virgaurea*: Morphological response to nutritional variation. Acta Phytoecologica Sinica 24: 46-51.

Shibaike H, Ishiguri Y, Kawano S (1996) Plastic responses to nutrient and light intensity gradients in populations of *Oxalis corniculata* L. (Oxalidaceae). Plant Species Biology 11: 213-223.

Slade AJ, Hutchings MJ (1987) An analysis of the costs and benefits of physiological integration between ramets in the clonal perennial herb *Glechoma hederacea*. Oecologia 73: 425-431.

Slade AJ, Hutchings MJ (1987) Clonal integration and plasticity in foraging behavior in *Glechoma hederacea*. Journal of Ecology 75: 1023-1036.

Slade AJ, Hutchings MJ (1987) The effects of light intensity on foraging in the clonal herb *Glechoma hederacea*. Journal of Ecology 75: 639-650.

Slade AJ, Hutchings MJ (1987) The effects of nutrient availability on foraging in the clonal herb *Glechoma hederacea*. Journal of Ecology 75: 95-112.

Stoll P, Egli P, Schmid B (1998) Plant foraging and rhizome growth patterns of *Solidago altissima* in response to mowing and fertilizer application. Journal of Ecology 86: 341-354.

Stuefer JF, Huber H (1998) Differential effects of light quantity and spectral light quality on growth, morphology and development of two stoloniferous *Potentilla* species. Oecologia 117: 1-8.

Sun XL, Niu JZ, Xu YF, Zhou H (2010) Long term water integration in interconnected ramets of stoloniferous grass, buffalograss. African Journal of Biotechnology 9: 5503-5510.

Sun XL, Niu JZ, Zhou H (2011) Buffalograss decreases ramet propagation in infertile patches to enhance interconnected ramet proliferation in fertile patches. Flora 206: 380-386.

Tao JP, Zhong ZC (2000) Morphological responses to different nutrient supply in the stoloniferous herb *Glechoma longituba*. Acta Ecologica Sinica 2: 207-211.

Thomas R, Hay M (2008) Adaptive variation in physiological traits underpinning stem elongation responses among nodally-rooting stoloniferous herbs. Evolutionary Ecology 22: 369-381.

Thomas RG, Hay MJM (2010) The role of nodal roots in prostrate clonal herbs: ‘Phalanx’ versus ‘guerrilla’. Evolutionary Ecology 24: 1489-1504.

van Kleunen M (2007) Adaptive genetic differentiation in life-history traits between populations of *Mimulus guttatus* with annual and perennial life-cycles. Evolutionary Ecology 21: 185-199.

Verburg RW, During HJ (1998) Vegetative propagation and sexual reproduction in the woodland understorey pseudo-annual *Circaea lutetiana* L. Plant Ecology 134: 211-224.

Vojtíšková L, Munzarová E, Votrubová O, Řihová A, Juřicová B (2004) Growth and biomass allocation of sweet flag (*Acorus calamus* L.) under different nutrient conditions. Hydrobiologia 518: 9-22.

Wang P, Lei JP, Li MH, Yu FH (2012) Spatial heterogeneity in light supply affects intraspecific competition of a stoloniferous clonal plant. PLoS ONE 7: e39105.

Wang YH (2005) The study on morphological plasticity of *Aeluropus littoralis* var. *sinensis* population in Songnen plain. Jilin: Northeast Normal University. 36 p.

Wijesinghe DK, Hutchings MJ (1996) Consequences of patchy distribution of light for the growth of the clonal herb *Glechoma hederacea*. Oikos 77: 137-145.

Wijesinghe DK, Whigham DF (2001) Nutrient foraging in woodland herbs: A comparison of three species of *Uvularia* (Liliaceae) with contrasting belowground morphologies. American Journal of Botany 88: 1071-1079.

Wolfer SR (2008) Clonal architecture and patch formation of *Potamogeton perfoliatus* L. in response to environmental conditions: Wageningen Universiteit. 120 p.

Wolfer SR, Straile D (2004) Spatio-temporal dynamics and plasticity of clonal architecture in *Potamogeton perfoliatus*. Aquatic Botany 78: 307-318.

Xing MJ (2010) The strategy of spacial expansion of clonal plant *Hierochloe glabra* Trin. under homogeneous and heterogeneous habitats. Jilin: Northeast Normal University. 40 p.

Xu CY, Schooler SS, Van Klinken RD (2012) Differential influence of clonal integration on morphological and growth responses to light in two invasive herbs. PLoS ONE 7: e35873.

Xu GF, Shen SC, Zhang FD, Li TL, Zhang YH (2013) Effect of soil-water conditions on survival rate and morphological plasticity of clonal plant *Mikania micrantha* H.B. Kunth. Scientia Agricultura Sinica 46: 3134-3141.

Xu KY, Ye WH, Li J, Li GM (2005) Phenotypic plasticity in response to soil nutrients in the invasive species *Alternanthera philoxeroides*. Ecology and Environment 14: 723-726.

Ye XH, Yu FH, Dong M (2006) A trade-off between guerrilla and phalanx growth forms in *Leymus secalinus* under different nutrient supplies. Annals of Botany 98: 187-191.

You WH, Yu D, Liu CH, Xie D, Xiong W (2013) Clonal integration facilitates invasiveness of the alien aquatic plant *Myriophyllum aquaticum* L. under heterogeneous water availability. Hydrobiologia 718: 27-39.

Yu FH, Dong M (2003) Effect of light intensity and nutrient availability on clonal growth and clonal morphology of the stoloniferous herb *Halerpestes ruthenica*. Acta Botanica Sinica 45: 408-416.

Yue CL, Chang J, Wang KH, Zhu YM (2004) Response of clonal growth in *Phyllostachys praecox* f. *prevernalis* to changing light intensity. Australian Journal of Botany 52: 171-174.

Yue CL, Wang KH, Zhu YM (2005) Morphological plasticity of clonal plant *Phyllostachys praecox* f. *prevemalis* (Poaceae) in response to nitrogen availability. Annales Botanici Fennici 42: 123-127.

Zhang CY, Yu FH, Chen YF, Dong M (2003) Phenotypic plasticity in response to the heterogeneous water supply in the rhizomatous grass species, *Calamagrostis epigejos* in the Mu Us Sandy Land of China. Acta Botanica Sinica 45: 1210-1217.

Zhang SM, Chen YF, Dong M (2000) Clonal plasticity in response to partial neutral shading in the stoloniferous herb *Potentilla reptans* var. *sericophylla*. Acta Botanica Sinica 42: 89-94.

Zhang YC, Zhang QY, Yirdaw E, Luo P, Wu N (2008) Clonal integration of *Fragaria orientalis* driven by contrasting water availability between adjacent patches. Botanical Studies 49: 373-383.

Zhao W, Chen SP, Lin GH (2008) Compensatory growth responses to clipping defoliation in *Leymus chinensis* (Poaceae) under nutrient addition and water deficiency conditions. Plant Ecology 196: 85-99.
