# Supplementary material for: Phylogenetic Meta-Analysis of the Functional Traits of Clonal Plants Foraging in Changing Environments
Source: PLoS One. 2014 Sep 12;9(9):e107114. doi: 10.1371/journal.pone.0107114 (PMC4162570; doi:10.1371/journal.pone.0107114)
Supplement: Appendix S3 — Phylogenetic tree, funnel plot and normal quantile plot. (DOC) [file pone.0107114.s003.doc]

**Phylogenetic tree:**

**Newick:**

((((((((((((((((((((((Agrostis_stolonifera:88.679245)Agrostis:88.679245,(Calamagrostis_canadensis:88.679245,Calamagrostis_epigejos:88.679245)Calamagrostis:88.679245,(Holcus_mollis:88.679245)Holcus:88.679245,(Microstegium_vimineum:88.679245)Microstegium:88.679245,(Phyllostachys_praecox_f._prevernalis:88.679245)Phyllostachys:88.679245,(Aeluropus_litoralis_var._sinensis:88.679245)Aeluropus:88.679245,(Agropyron_repens:88.679245)Agropyron:88.679245,(Buchloe_dactyloides:88.679245)Buchloe:88.679245,(Elymus_lanceolatus:88.679245,Elymus_repens:88.679245)Elymus:88.679245,(Hierochloe_glabra:88.679245)Hierochloe:88.679245,(Leymus_chinensis:88.679245,Leymus_secalinus:88.679245)Leymus:88.679245,(((((((((((Phragmites_australis:19.002695,Phragmites_communis:19.002695)phragmites:19.002695):19.002697):19.002693,(((((Zoysia_japonica:12.668464)zoysia:12.668464):12.668463):12.668465):12.668465):12.668461):12.668465):12.668465):12.668465):12.668465)pacc:12.668465):12.668457):12.668472):12.668457,(Psammochloa_villosa:88.679245)Psammochloa:88.679245,(Drepanostachyum_luodianense:88.679245)Drepanostachyum:88.679245,(Pleioblastus_maculata:88.679245)Pleioblastus:88.679245)poaceae:12.668457):12.668472):12.668457):12.668472,((((((Carex_montis-everestii:32.576050,Carex_arenaria:32.576050,Carex_disticha:32.576050,Carex_flacca:32.576050,Carex_hirta:32.576050)Carex:32.576050,(Cyperus_esculentus:32.576050)Cyperus:32.576050,(Schoenoplectus_americanus:32.576050)Schoenoplectus:32.576050,(Cladium_jamaicense:32.576050)Cladium:32.576050,(Eleocharis_cellulosa:32.576050)Eleocharis:32.576050)cyperaceae:32.576050):32.576050):32.576050):32.576050):32.576050):12.668457):12.668472,(((Typha_domingensis:63.342319)Typha:63.342319)typhaceae:63.342323):63.342316)poales:12.668442,((((((Eichhornia_crassipes:38.005390)Eichhornia:38.005390)pontederiaceae:38.005386):38.005394):38.005386)commelinales:38.005386):38.005386)commelinids:12.668488,(((((((Iris_japonica:34.838276)Iris:34.838276)iridaceae:34.838280):34.838272):34.838272):34.838287):34.838272)asparagales:34.838272):12.668457,((((((Uvularia_perfoliata:41.624950,Uvularia_sessilifolia:41.624950)Uvularia:41.624950)liliaceae:41.624954):41.624947):41.624954):41.624954)liliales:41.624954):12.668457):12.668457):12.668488,((((((((((Potamogeton_amplifolius:34.310425,Potamogeton_perfoliatus:34.310425)Potamogeton:34.310425)potamogetonaceae:34.310425):34.310425,((((Cymodocea_nodosa:27.448339)Cymodocea:27.448339,(Halodule_wrightii:27.448339)Halodule:27.448339)cymodoceaceae:27.448338):27.448341):27.448341):27.448334):27.448334):27.448349):27.448334):27.448349):27.448334)alismatales:27.448334):12.668457,(((Acorus_calamus:85.512131)Acorus:85.512131)acoraceae:85.512115)acorales:85.512146)monocots:12.668457,(((((((((((((((((Alternanthera_philoxeroides:31.865828)Alternanthera:31.865828)amaranthaceae:31.865826):31.865829):31.865822):31.865829):31.865829):31.865829)caryophyllales:31.865829,(((((((((Anagallis_tenella:38.037739)Anagallis:38.037739,(Lysimachia_nummularia:38.037739)Lysimachia:38.037739,(Trientalis_europaea:38.037739)Trientalis:38.037739)primulaceae:38.037735):38.037743):38.037735,((((((Gaultheria_shallon:27.169811)Gaultheria:27.169811)ericaceae:27.169815):27.169807):27.169815):27.169815):27.169815):27.169800):27.169815)ericales:27.169815,((((((((Convolvulus_arvensis:44.905663,Convolvulus_chilensis:44.905663)Convolvulus:44.905663,(Calystegia_silvatica:44.905663)Calystegia:44.905663)convolvulaceae:44.905655):44.905670)solanales:44.905655,((((((((Cymbalaria_muralis:48.427673)Cymbalaria:48.427673,(Mimulus_guttatus:48.427673)Mimulus:48.427673)scrophulariaceae:48.427673,(((((Glechoma_hirsuta:46.226414,Glechoma_longituba:46.226414)Glechoma:46.226414,(Lamiastrum_galeobdolon:46.226414)Lamiastrum:46.226414,(Ajuga_reptans:46.226414)Ajuga:46.226414,(Mentha_pulegium:46.226414)Mentha:46.226414,((((((glechoma_hederacea:13.207547):13.207547):13.207548):13.207546):13.207546):13.207550):13.207542)lamiaceae:13.207550):13.207542,((((Phyla_canescens:23.773584)Phyla:23.773584)verbenaceae:23.773586):23.773582):23.773582):13.207550):13.207550):13.207550):13.207535):13.207550):13.207550):13.207550)lamiales:13.207550):13.207535,((((((((Rubia_peregrina:26.415092)rubia:26.415092):26.415092):26.415092):26.415100):26.415085)rubioideae:26.415100)rubiaceae:26.415085,(((((Vinca_minor:35.220123)Vinca:35.220123)apocynaceae:35.220123):35.220123):35.220123):35.220123)gentianales:26.415100):13.207550)lamiids:13.207550,((((((((((((Hydrocotyle_vulgaris:22.641510)Hydrocotyle:22.641510)apiaceae:22.641510):22.641510):22.641510):22.641510):22.641510):22.641510)apiales:22.641510,((((Linnaea_borealis:40.754719)Linnaea:40.754719)caprifoliaceae:40.754715)dipsacales:40.754723):40.754715):22.641510):22.641510,((((((((((((((((((((((((Cirsium_dissectum:11.859838)cirsium:11.859838):11.859837):11.859840):11.859837):11.859837):11.859840):11.859840):11.859833):11.859840):11.859840):11.859833):11.859848):11.859833,(((((((((Ligularia_virgaurea:25.660376)ligularia:25.660376):25.660374):25.660378):25.660378,((((((((((((((((Solidago_altissima:7.547170,Solidago_canadensis:7.547170,Solidago_gigantea:7.547170)solidago:7.547170):7.547171):7.547169):7.547169):7.547173):7.547169):7.547169):7.547173):7.547165):7.547173):7.547173):7.547165):7.547173):7.547173):7.547165):7.547165):7.547180):7.547165):7.547165):7.547180):7.547165):7.547165):7.547180):7.547165):7.547165):7.547180,(Leptinella_dioica:101.886795)Leptinella:101.886795,(Symphyotrichum_lanceolatum:101.886795)Symphyotrichum:101.886795,(Mikania_micrantha:101.886795)Mikania:101.886795)asteraceae:7.547165):7.547165):7.547180):7.547165):7.547165)asterales:7.547180):7.547150)campanulids:7.547180):7.547180)ericales_to_asterales:7.547150)asterids:7.547180):7.547180):7.547150):7.547180,((((((((((Circaea_lutetiana:34.936092)Circaea:34.936092)onagraceae:34.936089):34.936096):34.936096)myrtales:34.936081):34.936096)malvids:34.936096,(((((((Duchesnea_indica:49.908703)Duchesnea:49.908703,(Fragaria_vesca:49.908703,Fragaria_chiloensis:49.908703,Fragaria_orientalis:49.908703)Fragaria:49.908703,(Potentilla_anglica:49.908703,Potentilla_anserina:49.908703,Potentilla_reptans:49.908703)Potentilla:49.908703,(Acaena_caesiiglauca:49.908703)Acaena:49.908703)rosaceae:49.908699)rosales:49.908707):49.908707,((((((((((((((((((((((((Trifolium_fragiferum:9.981741,Trifolium_repens:9.981741)trifolium:9.981741):9.981741):9.981741):9.981739):9.981743):9.981739):9.981743):9.981735)irlc:9.981743):9.981743):9.981743):9.981743):9.981735):9.981735):9.981750):9.981735):9.981735)papilionoideae:9.981750):9.981735):9.981735):9.981750)fabaceae:9.981735):9.981750)fabales:9.981735):9.981750,(((((((Oxalis_corniculata:32.440659)Oxalis:32.440659)oxalidaceae:32.440659):32.440659):32.440659)oxalidales:32.440659):32.440659)celastrales_to_malpighiales:32.440659):9.981720)fabids:9.981750):9.981750)rosids:9.981720,(((((((((Myriophyllum_aquaticum:29.945221)Myriophyllum:29.945221)haloragaceae:29.945221):29.945221):29.945221):29.945221):29.945221):29.945221):29.945221)saxifragales:29.945221):9.981750):7.547180)core_eudicots:7.547150)trochodendrales_to_asterales:7.547180)sabiales_to_asterales:7.547180,(((((((Halerpestes_ruthenica:42.452831)Halerpestes:42.452831,(Ranunculus_repens:42.452831)Ranunculus:42.452831)ranunculaceae:42.452827,((Podophyllum_peltatum:42.452831)Podophyllum:42.452831)berberidaceae:42.452827):42.452835):42.452835):42.452820):42.452850)ranunculales:42.452820)eudicots:7.547150)ceratophyllales_and_eudicots:7.547180)poales_to_asterales:7.547180)magnoliales_to_asterales:7.547150)austrobaileyales_to_asterales:7.547180)nymphaeales_to_asterales:7.547180)angiosperms:7.547150)seedplants:7.547170)euphyllophyte:1.000000;

**Funnel plot:**

**Normal Quantile plot:**

Standardized effect size

-5.98

-2.67

0.65

3.96

7.28

-2.95

-1.47

0.00

1.47

2.95

Normal quantile
