# Supplementary material for: Phylogenetic Meta-Analysis of the Functional Traits of Clonal Plants Foraging in Changing Environments
Source: PLoS One. 2014 Sep 12;9(9):e107114. doi: 10.1371/journal.pone.0107114 (PMC4162570; doi:10.1371/journal.pone.0107114)
Supplement: Appendix S4 — Results with the random-model in traditional meta-analysis versus PMA by phyloMeta v1.3. (DOC) [file pone.0107114.s004.doc]

**Appendix D:** The results with random-model in traditional meta-analysis (without phylogenetic information, TMA) vs. phylogenetic meta-analysis (with phylogenetic information, PMA) by software phyloMeta v1.3.

|  | **Light** | | | | | | | | |
| --- | --- | --- | --- | --- | --- | --- | --- | --- | --- |
|  | **TMA** | | | | **PMA** | | | | |
| **Trait Group** | N | *d+* | Var. | 95% CI | N | *d+* | Var. | 95% CI | |
| **Branching angle** | Q_b_ = 0.00, d.f. = 1, *p* > 0.05 | | | | Q_b_ = 0.00, d.f. = 1, *p* > 0.05 | | | | |
| All | 2, 6 | 0.55 | 0.08 | (-0.01, 1.11) | 2, 6 | **0.61** | **0.09** | | **(0.01, 1.20)** |
| Rhizomatous | -- | -- | -- | -- | -- | -- | -- | | -- |
| Stoloniferous | -- | -- | -- | -- | -- | -- | -- | | -- |
| Monopodial | -- | -- | -- | -- | -- | -- | -- | | -- |
| Sympodial | 2, 6 | 0.55 | 0.08 | (-0.01, 1.11) | 2, 6 | 0.00 | 0.08 | | (-0.56, 0.57) |
| **Branching intensity** | Q_b_ = 0.67, d.f. = 1, *p* > 0.05 | | | | Q_b_ = 32.87 d.f. = 1, ***p* <** **0.05** | | | | |
| All | 18, 85 | **1.19** | **0.09** | **(0.59, 1.78)** | 18, 85 | 0.76 | 0.17 | | (-0.04, 1.55) |
| Rhizomatous | 7, 31 | 0.64 | 0.22 | (-0.28, 1.56) | 7, 31 | 0.62 | 0.26 | | (-0.38, 1.62) |
| Stoloniferous | 11, 54 | **1.59** | **0.16** | **(0.80, 2.37)** | 11, 54 | **1.97** | **0.22** | | **(1.06, 2.89)** |
| Monopodial | 4, 30 | **1.44** | **0.43** | **(0.16, 2.73)** | 4, 30 | **1.70** | **0.46** | | **(0.38, 3.02)** |
| Sympodial | 14, 55 | **1.11** | **0.11** | **(0.47, 1.75)** | 14, 55 | **1.39** | **0.15** | | **(0.63, 2.15)** |
| **Spacer length** | Q_b_ = 2.55, d.f. = 1, *p* > 0.05 | | | | Q_b_ = 3.44, d.f. = 1, *p* > 0.05 | | | | |
| All | 27, 132 | -0.15 | 0.06 | (-0.65, 0.34) | 27, 132 | -0.30 | 0.15 | | (-1.05, 0.46) |
| Rhizomatous | 8, 42 | 0.37 | 0.23 | (-0.56, 1.30) | 8, 42 | -0.34 | 0.15 | | (-1.08, 0.41) |
| Stoloniferous | 19, 90 | -0.36 | 0.09 | (-0.94, 0.23) | 19, 90 | 0.80 | 0.34 | | (-0.34, 1.94) |
| Monopodial | 7, 27 | -0.62 | 0.23 | (-1.57, 0.32) | 7, 27 | 0.71 | 0.28 | | (-1.75, 0.33) |
| Sympodial | 20, 105 | 0.02 | 0.09 | (-0.56, 0.60) | 20, 105 | -0.24 | 0.15 | | (-1.00, 0.52) |
| **Specific spacer length** | Q_b_ = 0.00, d.f. = 1, *p* > 0.05 | | | | Q_b_ = 0.00, d.f. = 1, *p* > 0.05 | | | | |
| All | 3, 11 | -0.42 | 0.09 | (-0.99, 0.15) | 3, 11 | -0.66 | 0.17 | | (-1.48, 0.15) |
| Rhizomatous | 3, 11 | -0.42 | 0.09 | (-0.99, 0.15) | 3, 11 | -0.66 | 0.17 | | (-1.48, 0.15) |
| Stoloniferous | -- | -- | -- | -- | -- | -- | -- | | -- |
| Monopodial | -- | -- | -- | -- | -- | -- | -- | | -- |
| Sympodial | 3, 11 | -0.42 | 0.09 | (-0.99, 0.15) | 3, 11 | -0.66 | 0.17 | | (-1.48, 0.15) |
|  |  | | | | | | | | |
|  | **Nutrient** | | | | | | | | |
|  | **TMA** | | | | **PMA** | | | | |
| **Trait Group** | N | *d+* | Var. | 95% CI | N | *d+* | Var. | 95% CI | |
| **Branching angle** | Q_b_ = 0.00, d.f. = 1, *p* > 0.05 | | | | Q_b_ = 2.20, d.f. = 1, *p* > 0.05 | | | | |
| All | 3, 13 | -0.72 | 0.52 | (-2.14, 0.69) | 3, 13 | -0.61 | 0.55 | (-2.06, 0.84) | |
| Rhizomatous | 2, 10 | 0.04 | 0.04 | (-0.38, 0.45) | 2, 10 | 0.04 | 0.05 | (-0.40, 0.47) | |
| Stoloniferous | -- | -- | -- | -- | -- | -- | -- | -- | |
| Monopodial | -- | -- | -- | -- | -- | -- | -- | -- | |
| Sympodial | 3, 13 | -0.72 | 0.52 | (-2.14, 0.69) | 3, 13 | -0.68 | 0.53 | (-2.11, 0.75) | |
| **Branching intensity** | Q_b_ = 15.13, d.f. = 1, ***p* < 0.05** | | | | Q_b_ = 11.93, d.f. = 1, ***p* < 0.05** | | | | |
| All | 26, 106 | **1.02** | **0.04** | **(0.62, 1.41)** | 26, 106 | **0.59** | **0.09** | **(0.01, 1.18)** | |
| Rhizomatous | 13, 38 | 0.44 | 0.07 | (-0.09, 0.96) | 13, 38 | 0.66 | 0.12 | (-0.07, 1.34) | |
| Stoloniferous | 13, 68 | **1.77** | **0.09** | **(1.18, 2.37)** | 13, 68 | **2.31** | **0.14** | **(1.59, 3.03)** | |
| Monopodial | 6, 40 | **2.78** | **0.20** | **(1.90, 3.65)** | 6, 40 | **3.14** | **0.27** | **(2.12, 4.17)** | |
| Sympodial | 20, 66 | **0.63** | **0.03** | **(0.27, 0.99)** | 20, 66 | **0.88** | **0.06** | **(0.40, 1.36)** | |
| **Spacer length** | Q_b_ = 0.97, d.f. = 1, *p* > 0.05 | | | | Q_b_ = 1.20, d.f. = 1, *p* > 0.05 | | | | |
| All | 45, 490 | 0.21 | 0.01 | (-0.02, 0.44) | 45, 490 | 0.09 | 0.03 | (-0.27, 0.46) | |
| Rhizomatous | 28, 401 | 0.15 | 0.02 | (-0.14, 0.43) | 28, 401 | 0.03 | 0.04 | (-0.38, 0.44) | |
| Stoloniferous | 17, 89 | 0.32 | 0.04 | (-0.06, 0.70) | 17, 89 | -0.01 | 0.06 | (-0.47, 0.46) | |
| Monopodial | 5, 31 | 0.69 | 0.14 | (-0.03, 1.41) | 5, 31 | 0.31 | 0.16 | (-0.48, 1.10) | |
| Sympodial | 40, 459 | 0.16 | 0.02 | (-0.09, 0.40) | 40, 459 | 0.34 | 0.03 | (-0.004, 0.69) | |
| **Specific spacer length** | Q_b_ = 0.00, d.f. = 1, *p* > 0.05 | | | | Q_b_ = 0.00, d.f. = 1, *p* > 0.05 | | | | |
| All | 3, 17 | 0.02 | 0.06 | (-0.45, 0.49) | 3, 17 | -0.01 | 0.08 | (-0.58, 0.56) | |
| Rhizomatous | 3, 17 | 0.02 | 0.06 | (-0.45, 0.49) | 3, 17 | -0.001 | 0.07 | (-0.51, 0.51) | |
| Stoloniferous | -- | -- | -- | -- | -- | -- | -- | -- | |
| Monopodial | -- | -- | -- | -- | -- | -- | -- | -- | |
| Sympodial | 3, 17 | 0.02 | 0.06 | (-0.45, 0.49) | 3, 17 | -0.001 | 0.07 | (-0.51, 0.51) | |
|  |  |  |  |  |  |  |  |  | |
|  | **Water** | | | | | | | | |
|  | **TMA** | | | | **PMA** | | | | |
| **Trait Group** | N | *d+* | Var. | 95% CI | N | *d+* | Var. | 95% CI | |
| **Banching angle** | Q_b_ = 0.07, d.f. = 1, *p* > 0.05 | | | | Q_b_ = 0.52, d.f. = 1, *p* > 0.05 | | | | |
| All | -- | -- | -- | -- | -- | -- | -- | -- | |
| Rhizomatous | -- | -- | -- | -- | -- | -- | -- | -- | |
| Stoloniferous | -- | -- | -- | -- | -- | -- | -- | -- | |
| Monopodial | -- | -- | -- | -- | -- | -- | -- | -- | |
| Sympodial | -- | -- | -- | -- | -- | -- | -- | -- | |
| **Branching intensity** | Q_b_ = 0.3.49, d.f. = 1, *p* > 0.05 | | | | Q_b_ = 5.94 d.f. = 1, ***p* <** **0.05** | | | | |
| All | 5, 22 | **1.13** | **0.40** | **(-0.10, 2.37)** | 5, 22 | 1.22 | 0.52 | (-0.19, 2.63) | |
| Rhizomatous | -- | -- | -- | -- |  | 0.60 | 0.26 | (-0.40,1.59) | |
| Stoloniferous | 4, 20 | 0.66 | 0.19 | (-0.20, 1.51) | 4, 20 | 0.76 | 0.31 | (-0.34, 1.85) | |
| Monopodial | 2, 6 | 1.21 | 1.16 | (-0.90, 3.32) | 2, 6 | 1.21 | 1.16 | (0.90, 3.32) | |
| Sympodial | 3, 16 | 1.05 | 0.85 | (-0.76, 2.85) | 3, 16 | 1.06 | 0.88 | (-0.77, 2.89) | |
| **Spacer length** | Q_b_ = 3.51, d.f. = 1, *p* > 0.05 | | | | Q_b_ = 43.74, d.f. = 1, *p* < 0.05 | | | | |
| All | 7, 55 | 0.58 | 0.38 | (-0.63,1.79) | 7, 55 | 0.26 | 0.56 | (-1.21, 1.73) | |
| Rhizomatous | 4, 31 | 0.08 | 0.65 | (-1.50, 1.66) | 4, 31 | 0.08 | 0.65 | (-1.50, 1.66) | |
| Stoloniferous | 3, 24 | 1.3 | 0.93 | (-0.59, 3.19) | 3, 24 | 1.82 | 0.96 | (-0.10, 3.75) | |
| Monopodial | -- | -- | -- | -- | -- | -- | -- | -- | |
| Sympodial | 7, 55 | 0.55 | 0.33 | (-0.57, 1.67) | 7, 55 | 0.19 | 0.49 | (-1.19, 1.56) | |
| **Specific spacer length** | Q_b_ = 0.69, d.f. = 1, *p* > 0.05 | | | | Q_b_ = 1.03, d.f. = 1, *p* > 0.05 | | | | |
| All | -- | -- | -- | -- | -- | -- | -- | -- | |
| Rhizomatous | -- | -- | -- | -- | -- | -- | -- | -- | |
| Stoloniferous | -- | -- | -- | -- | -- | -- | -- | -- | |
| Monopodial | -- | -- | -- | -- | -- | -- | -- | -- | |
| Sympodial | -- | -- | -- | -- | -- | -- | -- | -- | |

*N: the first is number of species contained; the second is number of cases combined;

*d+*, Var. : Pooled effect sizes and variances ( Hedges’ d);

95% CI: 95% confidence intervals (CI);

--: No enough data available.
